# Changes in the folding landscape of the WW domain provide a molecular mechanism for an inherited genetic syndrome

**DOI:** 10.1038/srep30293

**Published:** 2016-07-26

**Authors:** Encarna Pucheta-Martinez, Nicola D’Amelio, Moreno Lelli, Jorge L. Martinez-Torrecuadrada, Marius Sudol, Giorgio Saladino, Francesco Luigi Gervasio

**Affiliations:** 1 Department of Chemistry, University College London, London WC1E 6BT, United Kingdom; 2 Research Institute of Structural and Molecular Biology, University College London, London WC1E 6BT, United Kingdom; 3 University of Florence, Department of Chemistry, Magnetic Resonance Center (CERM), 50019 Sesto Fiorentino (FI), Italy; 4 Crystallography and Protein Engineering Unit, Spanish National Cancer Research Centre (CNIO), C/Melchor Fernandez Almagro 3, 28029, Madrid, Spain; 5 Institute of Molecular and Cell Biology A*STAR, 61 Biopolis, Singapore 138673, Republic of Singapore; 6 Mechanobiology Institute, 5A Engineering Drive 1, Singapore 117411, Republic of Singapore; 7 National University of Singapore, Department of Physiology, The Yong Loo Li School of Medicine, 2 Medical Drive, Singapore 117597, Republic of Singapore

## Abstract

WW domains are small domains present in many human proteins with a wide array of functions and acting through the recognition of proline-rich sequences. The WW domain belonging to polyglutamine tract-binding protein 1 (PQBP1) is of particular interest due to its direct involvement in several X chromosome-linked intellectual disabilities, including Golabi-Ito-Hall (GIH) syndrome, where a single point mutation (Y65C) correlates with the development of the disease. The mutant cannot bind to its natural ligand WBP11, which regulates mRNA processing. In this work we use high-field high-resolution NMR and enhanced sampling molecular dynamics simulations to gain insight into the molecular causes the disease. We find that the wild type protein is partially unfolded exchanging among multiple beta-strand-like conformations in solution. The Y65C mutation further destabilizes the residual fold and primes the protein for the formation of a disulphide bridge, which could be at the origin of the loss of function.

The WW is the smallest domain found in proteins (about 40 amino-acids) with a variety of functions within the cell^1^,^2^,^3^. Just as the SH3 domain, it recognizes proline-rich regions in cognate proteins^4^. Its diverse and regulated localization within the cell (both in the nucleus and the cytoplasm) stresses the biological importance of WW domain-containing proteins and explains why signalling via WW domain complexes is implicated in several human diseases including muscular dystrophy, Alzheimer and Huntington diseases, Liddle’s syndrome of hypertension, cancer and X chromosome linked intellectual disabilities^2^,^5^,^6^,^7^,^8^,^9^,^10^. The Golabi-Ito-Hall (GIH) syndrome, in particular, is an X-chromosome linked disease caused by a missense mutation in the WW domain of the Polyglutamine Binding Protein 1 (PQBP1), which is widely expressed in various organs but enriched in the brain. The WW domain of PQBP1 mediates the interaction with the nucleocytoplasmic shuttling splicing factor SIPP1 (previously known as NpwBP and WBP11), which regulates mRNA processing and transcription^11^, by recognizing the proline-rich sequence of SIPP1^12^,^13^. Mutations of PQBP-1 have also been reported in several other X-chromosome-linked intellectual disability disorders (XLID) and progressive neuro-degenerative diseases^6^,^8^,^14^,^15^. Possible molecular causes linking WW mutations to the GIH syndrome have been investigated by Sudol and coworkers^11^. In their study, the authors observed a moderate loss of signaling in the GIH-causing Y65C mutant and suggested that the fold of the WW domain might be compromised by the mutation, with consequent loss of interaction with its partners in the splicing complex.

The fold of WW domains is in general well known, consisting of a stable, triple stranded beta sheet^16^,^17^,^18^,^19^,^20^,^21^,^22^,^23^,^24^,^25^,^26^,^27^,^28^,^29^,^30^,^31^,^32^,^33^,^34^. The solution NMR structures of several WW domains have been determined revealing a common fold but also different degrees of conformational stability. While in general the domain is remarkably well ordered^16^,^17^,^18^,^19^,^20^,^21^,^22^,^23^,^24^,^25^,^26^,^27^,^28^,^29^,^30^,^31^,^32^,^33^,^34^, in some cases it presents conformational exchange^19^,^21^,^32^. The structure has been also studied in the presence of a binding peptide which might stabilize the fold^17^,^19^,^28^,^30^,^31^,^32^. Recently, the X-ray structure of the C-terminus of PQBP1 has been determined in complex with spliceosomal protein U5-15kD^35^, showing how a YxxPxxVL motif in PQBP1 is recognized. The WW domain, however, was not included in the protein sequence. Here we investigate the underlying causes of the GIH disease by using a combination of high-field solution NMR and state-of-the-art enhanced sampling simulations to determine the effect of the Y65C mutation on the structure and dynamics of the WW domain of PQBP1.

## Results and Discussion

### The WW domain, from Poly-glutamine binding protein (PQBP), exchanges among different conformations in solution

**Figure 1:**
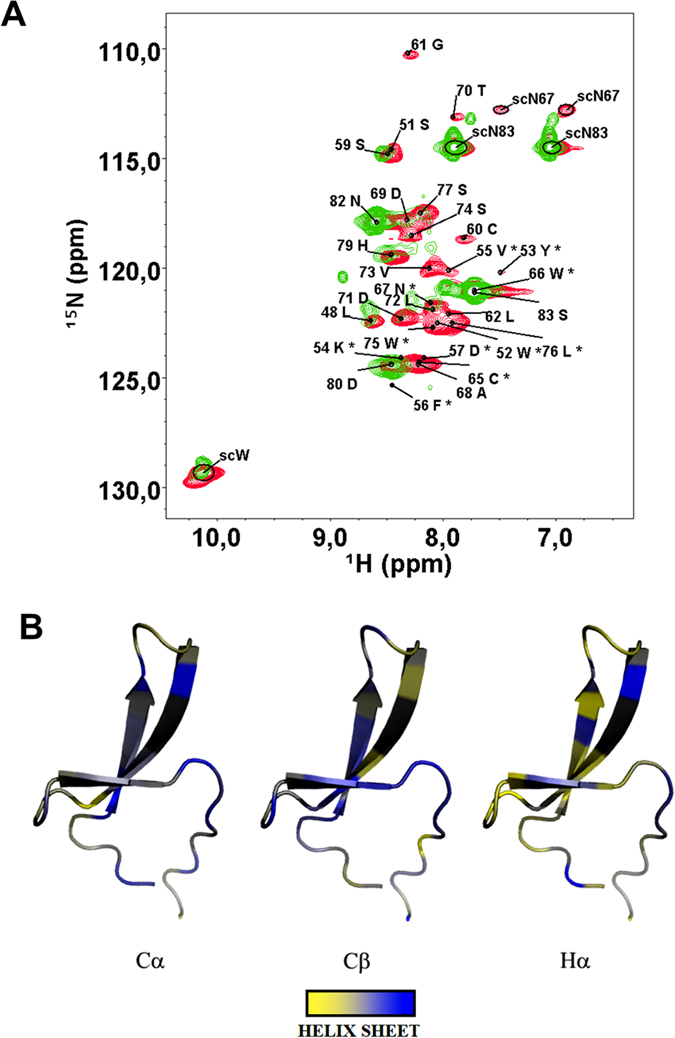
(**A**) ^1^H,^15^N-HSQC spectrum of WT WW domain (green) superimposed to the one of its Y65C mutant (red), for which also the assignment is shown (500 MHz of ^1^H Larmor frequency, T = 278 K). Residues labeled with a star (*) are tentatively assigned. (**B**) Pictorial representation of raw values of Chemical Shift Index onto the structural model of Y65C WW domain from PQBP. Beta strand-like structures (blue) are predominant. Deviations of opposite sign are displayed in yellow. Residues for which data are not available (unassigned) are colored in gray.

The ^1^H,^15^N HSQC spectrum of the PQBP1 WW domain (Fig. 1A), reveals that the protein exchanges among multiple conformations in solution, a behavior which has been reported for a few other WW domains^19^,^21^,^32^. The dispersion of signals in the proton dimension approaches the one expected for intrinsically disordered proteins. However, the large line-width is not expected for a random coil behavior. Despite the small size of the protein and the use of a doubly-labeled sample, the assignment of signals was particularly demanding (see Table S1 in Supporting Information). Excluding formation of large aggregates as a possible cause (the protein is purified by size exclusion chromatography, yielding one single peak consistent with a monomer) such extreme broadening could be explained by the presence of conformational exchange in the micro to milliseconds time scales. Severe line broadening is indicating the presence of dynamic exchange approaching the intermediate regime in the NMR time scale. This is observed when the difference in the frequency shifts of the exchanging resonances is comparable with the exchange rate. Thus changing pH, temperature (that affect the exchange rates) or changing the magnetic field (that scales the difference in the frequency shifts) could help in displacing the intermediate exchange regime towards a slow or a fast exchange regime that has narrower NMR line-width. We tried to modify the experimental conditions in order to get sharper lines for a detailed structural determination based on NOE analysis. Unfortunately the quality of the spectrum does not improve when the pH is lowered from 7.4 to 6.5 or by changing NMR field (500, 600, 700 and even 1000 MHz). Also the temperature has very little effect. The spectrum of the WT slightly improves at 313 K but the line-width remains so broad that a complete assignment is challenging. This is probably due to the low stability of the native fold combined with a broad energy basin, which might lead to a conformational exchange between various structures.

NMR allows the detection of secondary structure elements residue by residue even in the presence of conformational exchange. This is achieved by measuring the chemical shift of backbone carbon atoms (carbonyl, carbons in positions α and β but also Hα), whose deviations from the tabulated random coil values, is predictive of alpha-helices or beta-strands character^36^. The accuracy of the Chemical Shift Index (CSI)^36^ has been recently improved to 90%^37^. In cases of fast exchange, the value of the deviation is expected to be a population weighted average of the deviations expected for each structure. This decreases the absolute value of the CSI but it can still give an indication of the most populated secondary structure conformations in solution. In the present case, the calculated deviations are compatible with a beta fold for both the wild type (WT) and the mutant (Fig. 1B). Among the three predictors (Cα, Cβ and Hα), Cβ is the most reliable in detecting beta-strands. According to its values, beta structure is present where expected from the canonical WW fold, however it is not very stable and undergoes a significant conformational exchange dynamics. Similar conclusions have been reached by circular dichroism^38^. The presence of beta structures in the folded ensemble of the WW is expected, as the typical fold for this domain consists in a triple stranded beta sheet both in crystal structures and in solution^16^,^17^,^18^,^19^,^20^,^21^,^22^,^23^,^24^,^25^,^26^,^27^,^28^,^29^,^30^,^31^,^32^,^33^,^34^. A NMR-based model of our specific amino-acid sequence also yields a similar fold (Fig. 1B). The hydrophobic core consisting of two tryptophan residues (giving the name to the domain) is well packed with other conserved hydrophobic residues (Y65, N67, P78)^11^.

**Figure 2:**
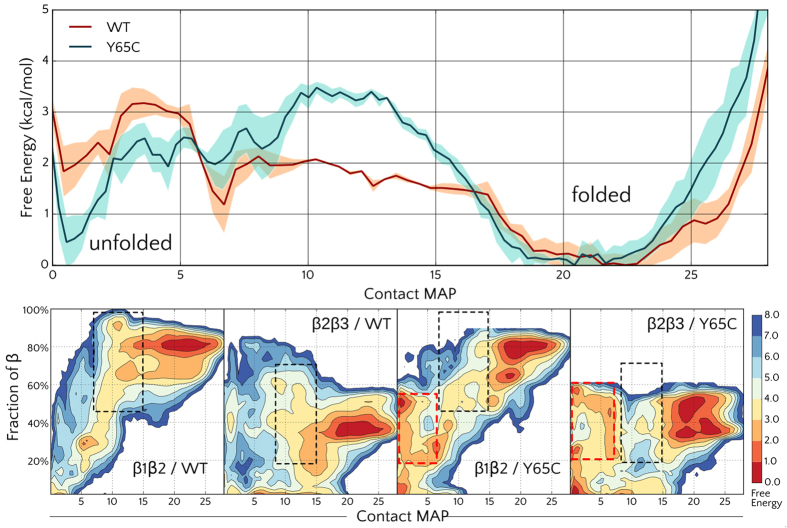
(**Top**) Free Energy profile of the WW domain WT and of the Y65C mutant as a function of the native contact map (CMAP); the shaded region represents the error. (**Bottom**) Projection of the WT and Y65C folding free energy profile along the contact map and the fraction of β structure of the first (β1β2) and second (β2β3) hairpins. The β1β2 element appears to be the most stable and is well structured also in partially folded conformations (black dotted boxes, CMAP between 10 and 15). The mutant partially folded conformations however, appear less structured, and misfolded conformations (with low CMAP and some residual β structure) appear (red boixes).

In order to explore the folding mechanism and better understand the reasons for the intrinsic instability of the fold, we performed enhanced sampling simulations with Parallel Tempering Metadynamics (PTMetaD)^39^ of both the wild-type and the Y65C mutant. Since the timescales needed to observe folding phenomena are frequently beyond the current capabilities of standard molecular dynamics (MD) simulations, enhanced-sampling algorithms are often used to study protein folding with atomistic models. Metadynamics-based approaches are among the most successful enhanced sampling methods and have been extensively used to study the folding free energy of small proteins^39^,^40^,^41^,^42^,^43^. Taking advantage of the recently developed well-tempered ensemble (WTE) approach^44^, we were able to use a more limited amount of replicas, namely 4, in the 300–400 K temperature range. The folding free energy landscape for the WT protein as a function of the selected collective variables (CV) is reported in Fig. 2A. The WT protein reported a folding free energy ΔG^U→F^ around −2.0 kcal/mol, compatible with the reported melting temperature^38^ of 45 °C. A broad main minimum is observed for the folded structure in the free energy (FE) profile, suggesting that some degree of heterogeneity is present within the folded state itself. Several intermediate states where either the first (β1β2) or the second (β2β3) hairpins are formed were also observed with energies 1-2 kcal/mol higher than the folded state. Molecular simulations thus give an explanation of the intrinsic instability (values of ΔG^U→F^ in small folded proteins, are commonly between −5 and −15 kcal/mol) and provide a structural view of the interchanging conformers.

### The WW domain Y65C mutant appears to be more disordered than the WT protein

The folding landscape of the WW Y65C mutant shares many similarities with the WT one, but the stability of the folded state is even lower, with a ΔG^U→F^ around −0.5 kcal/mol (Fig. 2A). The folding barrier is instead predicted to be higher (3.5 kcal/mol). The free energy profiles projected along the β1β2 contacts (Fig. 2B) show that, in addition to a stabilization of the unfolded structures, the Y65C considerably destabilized partially folded intermediates, where the β1β2 or β1β2 hairpins are formed. Indeed, a lower stability of the mutant is expected as the mutation destabilizes the well conserved hydrophobic core involving residues W52, Y65, N67 and P78^11^.

This is in agreement with the ^1^H,^15^N HSQC spectrum, whose signals are sharper, indicating higher flexibility, and with previous data^38^ based on far and near UV CD spectra, reporting a destabilizing effect of the mutation. The authors of Ref. 38 also found a more cooperative folding for the WT, regardless the similar melting temperature (45 °C).

The sharper peaks and better quality of the spectrum allowed us to record a 3D ^1^H,^15^N-NOESY-HSQC spectrum with good quality cross peaks. In well-folded proteins, the inter-proton Nuclear Overhauser Effect (NOE) is a major source of structural restrains to obtain molecular structures in solution by NMR. However, the accuracy of the distance estimated by NOE is strongly reduced in presence of conformational exchange dynamics. The changes in the inter-proton distances averages the NOE and thus the intensity of the NOESY cross-peak is not anymore directly related to the a distance value. In many cases, the amplitude of the conformational dynamics brings the inter-proton distances to values where the NOE is negligible, with a consequent weakening of NOESY cross-peaks, especially for long-range inter-proton contacts. For this reason, the absence of long-range NOESY cross-peaks observed in the 3D ^1^H,^15^N NOESY-HSQC spectrum of WW Y65C provides further evidence of an extended conformational dynamics. The sharper signals in the Y65C mutant HSQC are suggestive of a different dynamical regime with respect to the wild-type; it is possible that a higher flexibility in the Y65C mutant draws the dynamics toward a fast exchange in the NMR timescale with narrower lines. However, it should be noted that our data are not consistent with an intrinsically disordered protein. Extensive broadening is still present in the spectra of the mutant; and both the Chemical Shift Index (Fig. 1B) and simulations (Fig. 2), indicate the presence of beta structures in the ensemble. Furthermore, a dramatic difference of signal intensity is observed in both proteins along the sequence (see Figure S1 in SI). This is more consistent with a model where some residual structured elements are in conformational exchange among different forms, rather than the hypothesis of a random coil peptide, that would display narrow and intense lines for almost all its amino-acids.

### The Y65C mutant of WW domain easily oxidizes *in vitro*

The destabilization of the WW fold could, in principle, impair the binding of the WW molecular partners and be, on its own, a direct cause of the GIH syndrome. However, the oxidation of Cys_65_ in the Y65C mutant has also been proposed as the molecular cause of a loss of function in PQBP1^11^. *In vitro*, the protein dimerizes^38^, this might be due to the formation of an intermolecular disulphide bridge.

**Figure 3:**
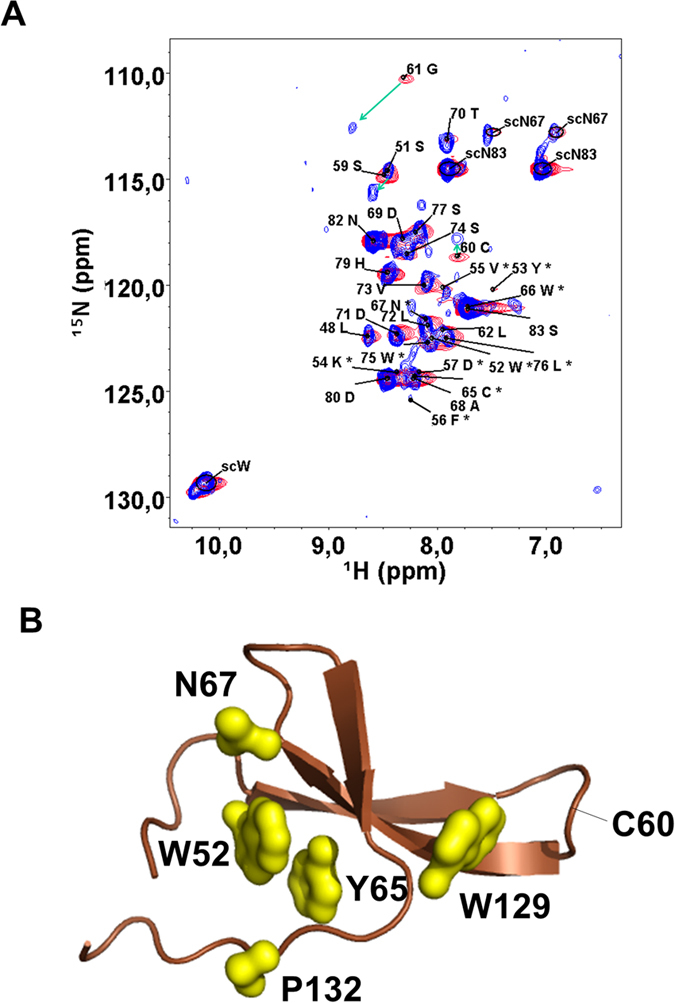
(**A**) ^1^H,^15^NHSQC spectrum of oxidized Y65C WW domain (blue, 500 MHz, 278 K) superimposed to the one of its reduced form (red). The assignment refers to the oxidized form. Green arrows indicate large chemical shift variations. (**B**) The hydrophobic core is disrupted by Y65C.

Indeed, our data show that the purified Y65C mutant of the WW domain is a soluble protein where Cys_60_ is clearly oxidized. This is unambiguously demonstrated by the chemical shift of Cys_60_ β-carbon, which is resonating at 41.6 ppm (the expected shift in reduced cysteines is around 30 ppm)^45^ The oxidation state of Cys_65_ is less evident because of its very weak amide signal, which makes possible only a tentative assignment. As Cys_60_ is in a loop (see the structure in Fig. 3B) and is directly connected to a flexible glycine residue, its involvement in inter-molecular disulphide bonds cannot be excluded, but this bond should also be formed in the WT, and we do not find any evidence for that. Addition of DTT promptly reduces Cys_60_ (Fig. 3A) and changes in the spectrum are limited to nearby residues (Gly_59_ and Ser_61_). A disulphide bond connecting two cysteines inter-molecularly is consistent with the limited changes observed in the ^1^H,^15^N-HSQC spectrum upon reduction. However, albeit small variations generally imply little change in structure, a partial loss of folding could be masked in the ensemble of conformational dynamics. Moreover, at variance with the WT, the mutant elutes as two peaks compatible with a monomer and a dimer, difficult to separate. In the NMR spectrum of the purified protein only the oxidized form is visible.

**Figure 4:**
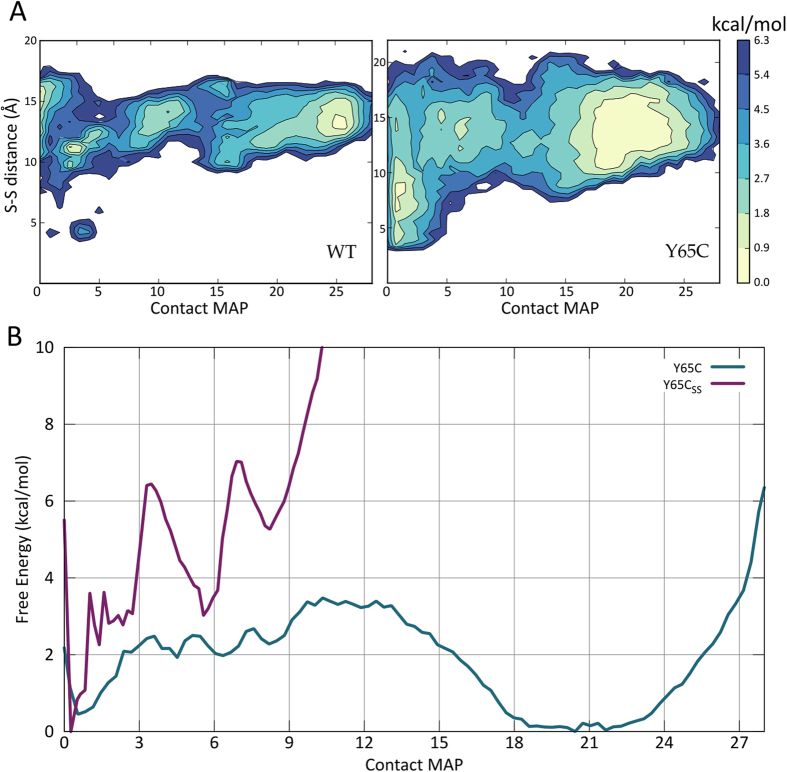
(**A**) Reweight of the Free Energy along the distance between the sulfur atoms of residues Cys60 and Cys65 (Tyr65 in the WT). The population of structures with a low distance between the two residues is increased in the Y65C mutant, with values as low as 4 Å in the unfolded state (CMAP < 5) of both proteins. (**B**) Free Energy of the Y65C mutant with and without the disulphide bridge between Cys60 and Cys65 as a function of the contact map.

We thus cannot exclude the formation of an intramolecular disuphide bond in competition with the intermolecular one *in vitro*. In cells, however, the latter would have a stronger biological meaning, since the domain is part of a much larger protein in the cell. A way to further address this question is to ascertain if the formation of an intramolecular disulphide bridge is compatible with the proposed folding ensemble. To assess the WW propensity to form structures compatible with the formation of the disulphide bridge, we re-weighted^46^ the FE profiles along the distance between the Cβ of residues Cys_60_ and Cys_65_ (Tyr_65_ in the WT). A more heterogeneous ensemble of distances was observed for the mutant, revealing an increased flexibility of the WW domain (Fig. 4).

Interestingly, structures compatible with the formation of a disulphide bond (around 4 Å) were clearly observed in the simulations. The structures with a short Cys60-X65 distance are considerably more populated in the mutant. However, short distances where only observed in the unfolded ensemble, suggesting that residues 60 and 65 are not able to approach one another when the protein is correctly folded. Indeed, the CD spectra of the mutant indicate a lower content in beta sheet with respect to the WT protein^38^, corroborating the hypothesis that the intramolecular disuplhide bridge stabilizes a partially different fold. For further proof, we extracted a structure with low Cys_60_-Cys_65_ distance from the free energy landscape of the Y65C mutant and tested the effect of the formation of a disulphide bridge in-silico. We performed a new PTMetaD with the same setup of the previous simulations and obtained a new folding profile (Fig. 4B). The protein explored several unfolded conformations and formed, transiently, the β1β2 sheet, but was never able to adopt the native fold.

## Conclusions

Understanding the folding behavior of the WW domain in solution may be essential to unravel the molecular causes of the GIH syndrome^11^,^38^. The WW domain from PQBP1 appears to have an unstable and disordered fold, at variance with most other WW domain structures determined so far in solution^16^,^17^,^18^,^19^,^20^,^21^,^22^,^23^,^24^,^25^,^26^,^27^,^28^,^29^,^30^,^31^,^32^,^33^,^34^. Although uncommon, conformational exchange between open and closed forms have been reported for other WW domains^19^,^21^,^32^.

The Y65C mutation of PQBP1 affects the hydrophobic core of the protein and may make it more prone to oxidation quenching the interaction with its biological partner. The domain, however, does not have the characteristic of an intrinsically disordered protein. NMR data are consistent with extensive exchange in the micro to milliseconds time scale between random coil and beta structures, and the chemical shift index reveals a significant amount of beta-sheet character. The hydrophobic residues W52, N67, Y65, W75 and P78 form a stable core, allowing the formation of three antiparallel beta-strands. Parallel Tempering Metadynamics explains the low stability of such fold, estimating a folding free energy around −2.0 kcal/mol. These values are in good agreement with thermal unfolding data, which report a melting temperature of 45 °C^38^. Mutation of Y65C is expected to destabilize significantly the residual structure of the protein, as the mutated tyrosine is right in the center of the hydrophobic core. Indeed, parallel Tempering Metadynamics predicts an even lower folding free energy (around −0.5 kcal/mol), while thermal unfolding suggests a more cooperative unfolding process with similar melting temperature^38^. Consistently, both CD^38^ and NMR suggest increased disorder and a larger contribution from random coil conformations reflected in a narrower line-width.

Conformational variability is essential for the accessibility of cysteine residues and therefore for their oxidation. The mutant tends to forms dimers *in vitro*^38^. Our experimental data are consistent with the presence of dimers but also monomers. In our NMR spectra, line broadening prevents the observations of the more structured residues of the protein, including Cys_65_. However, the signal of Cys_60_ (located in a loop of the structural model) is well visible and clearly oxidized. This allowed us to show that, unlike the WT protein, the mutant is able to form disulphide bridges through Cys_60_. We cannot exclude the formation of both an intramolecular and intermolecular disuplhide bridge. But since Cys_60_ is exposed in both the WT and the mutant, in the WT it does not tend to oxidize, and in cells the WW domain is part of a large multi-domain protein, we propose that the biologically relevant disulphide bridge is formed intra-molecularly, as shown in the simulations. Indeed, Parallel Tempering Metadynamics provides a clear picture of the folding and misfolding process, showing that not only an intramolecular bridge can be formed but it would also affect the folding landscape. The model accounts for both the increased disorder caused by the mutant and the large linewidth observed after oxidation.

Reversible oxidation of cysteine has been reported in physiological^47^ and pathological conditions^11^,^47^. As the formation of an intramolecular disulphide bridge would prevent the binding of the regulatory WW domain to its molecular partner, our model provides a new example of the role of cysteine oxidation and a plausible molecular mechanism at the basis of the GIH disease.

## Methods Summary

NMR experiments were performed on two different constructs of the WW domain of the PQBP1: a 37 amino acid long construct with sequence GLPPSWYKVFDPSCGLPY Y WNADTDLVSWLSPHDPNS and the corresponding Y65C (Y19C) mutant. NMR spectra were recorded on 0.5 mM samples at 278 K in 20 mM phosphate buffer at pH 6.5, 0.17 mM NaCl on Bruker Avance 500 MHz, 600 MHz, 700 MHz and 1000 MHz spectrometers. The simulations were performed with the CHARMM22* force field^48^ and explicit TIP3P water molecules^49^ and the PT-MetaD^39^,^50^,^51^ enhanced-sampling algorithm, using the software Gromacs-4.5.5 52 combined with the PLUMED^53^ plugin. The simulations were run until a satisfactory convergence of the free energy reconstruction was reached (650 ns per replica)^54^. Full materials and methods are reported in the SI.

## Additional Information

**How to cite this article**: Pucheta-Martinez, E. *et al.* Changes in the folding landscape of the WW domain provide a molecular mechanism for an inherited genetic syndrome. *Sci. Rep.* **6**, 30293; doi: 10.1038/srep30293 (2016).

## Supplementary Material

Supplementary Information
