# Supplementary material for: Changes in the folding landscape of the WW domain provide a molecular mechanism for an inherited genetic syndrome
Source: Sci Rep. 2016 Jul 26;6:30293. doi: 10.1038/srep30293 (PMC4960638; doi:10.1038/srep30293)
Supplement: Supplementary Information [file srep30293-s1.pdf]

Supplementary Information for: Changes in the folding landscape of the WW domain provide a molecular mechanism for an inherited genetic syndrome

Encarna Pucheta-Martinez+, Nicola D'Amelio+, Moreno Lelli, Jorge L. Martinez-Torrecuadrada, Marius Sudol, Giorgio Saladino and Francesco L. Gervasio**

Materials and Methods

Cloning, Expression and Purification. The WW domain was cloned in 2 different constructs: a 37 amino acid long construct with sequence GLPP SWYKVFDPSCGLPY Y WNADT DLVSWLSPHD PNS taken from the PQBP (poliglutamate binding protein)(MW = 4225.6 g/mol), and a 37 aminoacid long construct with the pathological mutation Y65C (position 19 in the sequence) GLPP SWYKVFDPSCGLPY C WNADT DLVSWLSPHD PNS (MW = 4165.6 g/mol). WW domain genes were cloned in pGEX vectors with resistance to Ampicillin and linked to GST protein (for protein purification) through a linker containing a TEV site (in order to free the WW domain from GST). Vectors were transformed into BL21 (DE3) cell lines. Colonies were amplified in 20 ml of Luria Broth (LB) medium, containing 20 mL of ampicillin (100 mg/mL). Cultures were grown at 37 °C and 220 rpm overnight. For labeled samples 10 mL of each culture grown overnight, were spun at 4000 rpm and their pellets were resuspended in 1 L of minimal medium (300 mL of a water solution containing 2.25 g $Na_2HPO_42H_2O$, 1.8 g KH_2PO_4, 0.15 g NaCl,0.075 g $MgSO_47H_2O$, 4.2 mg $CaCl_22H_2O$ were autoclaved and added with 10 mL 0.1 mg/mL Biothine, 3 mL 1% thiamine, 1 mL 100 mg/mL Ampicillin, 3.0 g of ^{13}C-Glucose, 1g ^{15}N-NH_4Cl. The final volume was brought to 1.0 L with water). For unlabelled proteins 10 mL were diluted to 1L of LB. The solutions were placed in incubator at 37 °C and 220 rpm. When the optical density (OD) reached values of 0.7-0.8, 200 mL of IPTG 1.0 M were added to each liter. Flasks were then transferred to an incubator at 20 °C and shaken at 220 rpm overnight (protein expression at 25 °C was demonstrated to cause protein degradation where GST spontaneously separates from the WW-domain). The solutions were then spun for 15 minutes at 4000 rpm. Pellets were stored at 20 or immediately processed. Pellets were resuspended in 50 mL of GST Buffer A (20 mM sodium phosphate pH 7.3, 0.15 M NaCl), using a container with ice. The lysate was then added with 18 mL benzonase, 7 mL lysozyme (30KU), and protease inhibitors mix. The solution was left at room temperature for 10 minutes, under mild shaking before being sonicated. The lysate was finally spun at 25000 rpm. GST 5 mL columns were equilibrated with GST buffer A and the sample was eluted introducing buffer GST B (50 mM TRISHCl pH 8, 10 mM reduced glutathion) without gradient. Samples were buffer exchanged by a 15 ml centricon with a cutoff of 10 KDa. The new buffer (TRISHCL 50 mM at pH 8, EDTA 0.5 mM e DTT 0.5 mM) is ideal for TEV cleavage activity. In the last concentration step, each sample was brought to 1.5 mL and added with 300 mL of TEV 0.5 mg/mL. The volume was brought to 2.0 mL with the same buffer and left at room temperature overnight. In case the mutant precipitates overnight, it can be resuspended by addition of DTT. Each 2.0 mL sample was introduced in S75 column (120 mL bed volume) equilibrated with PBS diluted 10 folds and DTT 0.5 mM, at a rate of 1 mL/min. While the cut GST appears invariably at about 5560 mL, different constructs appear at different volumes with an intensity of about 1/21/3: WW78S appears between 110 mL and 115 mL (right after the salts and partially overlapping with them), WW79S: between 95 and 110 mL. Samples were concentrated and buffer exchanged by 15 mL 3KDa cutoff centricon. The new buffer is a 20 mM phosphate solution at pH 6.5, 17 mM NaCl. Protein concentration can be checked via nanodrop using the following extinction coefficients at 280 nm: WW78S: 20970 $M^{-1}cm^{-1}$ (Abs 1 g/L = 4.963), WW79S: 19480 $M^{-1}cm^{-1}$ with cysteines reduced, 19605 oxidized (Abs 1 g/L = 4.676 or 4.706).

NMR spectra. Spectra were recorded typically on samples 0.5 mM at 278 K in 20 mM phosphate buffer at pH 6.5, 0.17mM NaCl. NMR spectra were acquired on Bruker Avance 500 MHz, 600 MHz, 700 MHz and 1000 MHz. NMR assignment was accomplished by using standard NMR sequences for ^{1}H,^{15}N-HSQC, ^{1}H,^{13}C-HSQC and 3D HNCA, HNCACB, ^{1}H,^{1}H-NOESY, ^{1}H,^{15}N-NOESYHSQC. NOESY mixing time was 200 ms. Spectra in the figures were recorded at 500 MHz. The HSQC pulse sequence included sensitivity enhancement. Spectra were recorded with a relaxation delay of 1 sec and 256 scans.

Simulations. The initial structure of the WW domain was obtained by homology modeling using the Swiss-Model Server. We used the CHARMM22* force field[1] with explicit TIP3P water molecules[2]. This force field has been developed and extensively tested to reproduce the folding behavior of small proteins and has been found to agree with NMR-derived experimental parameters in a previous study on the folding of a similar WW domain[3]. All simulations were run using Gromacs 4.5.5[4] combined with the PLUMED[5] plugin. The systems were minimized with 10000 steps of conjugated gradient and then equilibrated in the NPT ensemble for 10 ns at the pressure of 1 atm. The PT-MetaD simulations were performed until convergence of the free energy surfaces, for a total of 650 ns. The PME algorithm was used for electrostatic interactions with a cut-off of 1.2 nm. A single cut-off of 1.2 nm was used for Van der Waals interactions. Temperature coupling was done with the V-rescale algorithm[6]. According to the Well Tempered Ensemble (WTE) approach[7], a PT-MetaD using the potential energy as CV was performed until convergence (approx. 50 ns), using a bias factor of 100.0 and a σ of 1000 kJ/mol, in the NVT ensemble. These values were the optimal setup to obtain a uniform acceptance ratio of 25-30% across 4 replicas, with temperature in the range 300-400 K. After this initial simulation, the bias on the potential energy was written to file and maintained constant during the following step. For the PT-MetaD in the WTE, we used the secondary structure CV by Pietrucci et al.[8] and a contact map involving all the native interaction. These CV were chosen to monitor both the overall fold of the WW domain and the secondary structure of the β1β2 and β2β3 hairpins. The bias factor was set to 8.0 and the gaussians height to 1.0 kJ/mol, with a deposition rate of 1/2000 steps. The gaussians width was set to 0.1 for the contact map variable and to 0.05 for the secondary structure CV. The error on the free energy was calculated from the difference between the profile obtained summing the deposited gaussians and the one obtained with time-independent estimator developed by Tiwary and Parrinello[9]. This error was found to be always equal or larger that the error due to convergence in the last 200 ns.

Table S1. ^{1}H, ^{15}N, and ^{13}C backbone assignment of Y65C mutant of WWdomain from PQBP1.
Chemical shifts were referenced to internal DSS. Proteins concentration was 0.5 mM in 20 mM phosphate buffer at pH 6.5, 0.17mM NaCl. T=278 K. Residues highlighter with a star are temptatively assigned. Amide assignment of the *wt* protein and the oxidized form of the mutant are also reported.

Y65C Mutant reduced						Y65 oxidized		Wt WW domain	
Res	HN	N	Cα	Cβ	Hα	HN	N	HN	N
gly47	-	-	43.0	-	3.81	-	-	-	-
leu48	8.64	122.4	53.0	41.4	4.61	8.64	122.4	-	-
pro49	-	-	-	-	-	-	-	-	-
pro50	-	-	63.2	32.0	4.26	-	-	-	-
ser51	8.46	114.6	59.4	63.3	4.35	8.46	114.6	-	-
trp52*	8.05	122.5	53.2	29.5	4.64	8.05	122.5	-	-
tyr53*	7.49	120.2	53.2	38.8	4.32	-	-	-	-
lys54*	8.37	124.1	56.0	33.2	4.18	-	-	-	-
val55*	7.95	120.1	62.3	32.4	4.17	7.95	120.1	-	-
phe56*	8.32	125.5	57.8	39.2	4.31	8.32	125.5	-	-
asp57*	8.17	124.1	50.6	41.1	4.82	8.17	124.1	-	-
pro58	-	-	63.7	-	4.26	-	-	-	-
ser59	8.49	114.8	59.6	63.3	4.34	8.60	115.6	8.53	114.7
cys60	7.84	118.7	58.9	28.1	4.49	7.82	117.7	7.72	118.2
gly61	8.31	110.2	45.3	-	3.97	8.78	112.5	8.35	109.6
leu62	7.95	122.1	52.7	41.4	4.51	7.95	122.1	-	-
pro63	-	-	-	-	-	-	-	-	-
tyr64	-	-	-	-	4.38	-	-	-	-
cys65*	8.21	124.3	57.7	28.0	4.50	8.21	124.3	-	-

trp66*	7.72	121.0	57.3	29.5	4.64	7.72	121.0	-	-
asn67*	8.11	121.6	57.7	39.4	-	8.11	121.6	-	-
ala68	8.22	124.4	53.0	19.0	3.97	8.22	124.4	-	-
asp69	8.32	117.8	54.8	40.7	4.52	8.32	117.8	8.32	117.8
thr70	7.91	113.1	62.1	69.5	4.22	7.91	113.1	7.87	113.0
asp71	8.37	122.3	54.5	40.8	4.55	8.37	122.3	8.35	122.2
leu72	8.09	121.9	55.3	42.6	4.29	8.09	121.9	-	-
val73	8.12	120.0	62.7	32.5	4.02	8.12	120.0	-	-
ser74	8.28	118.5	58.4	63.5	4.38	8.28	118.5	-	-
trp75*	8.09	122.7	54.5	29.5	4.64	8.09	122.7	-	-
leu76*	7.92	122.5	54.6	42.5	4.29	7.92	122.5	-	-
ser77	8.20	117.5	56.1	63.3	4.56	8.20	117.5	8.18	117.5
pro78	-	-	63.1	31.9	4.41	-	-	-	-
his79	8.46	119.4	55.5	30.0	4.54	8.46	119.4	8.51	119.3
asp80	8.46	124.4	51.6	41.1	4.75	8.46	124.4	8.46	124.4
pro81	-	-	63.5	32.1	4.41	-	-	-	-
asn82	8.59	117.9	53.2	38.8	4.78	8.59	117.9	8.60	117.8
ser83	7.72	121.1	60.1	64.8	4.26	7.72	121.1	7.73	121.0

Figure S1. Amide proton signal intensity in $^{1}H,^{15}N$ -HSQC NMR spectrum, qualitatively displayed as backbone thickness and color (increasing from yellow to red) onto the structural model of Y65C mutant of WW domain from PQBP1. Data refer to the reduced mutant.

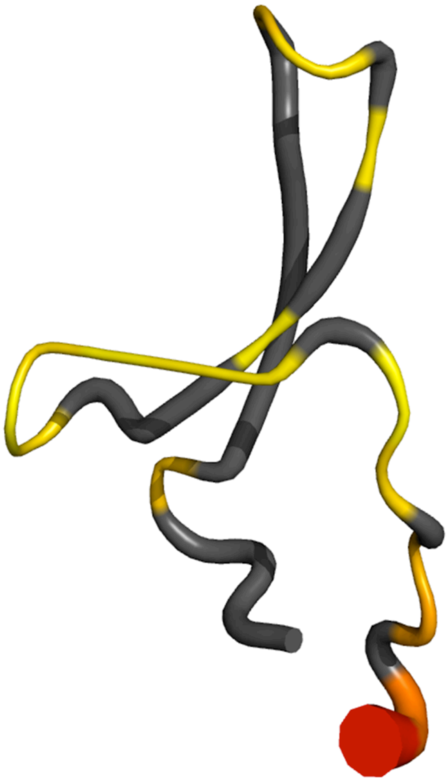

References

1. Piana, S., Lindorff-Larsen, K. & Shaw, D. E. How robust are protein folding simulations with respect to force field parameterization? *Biophys. J.* **100,** L47–9 (2011).

2. Jorgensen, W. L. Transferable intermolecular potential functions for water, alcohols, and ethers. Application to liquid water. *J Am Chem Soc* **103,** 335–340 (1981).

3. Lindorff-larsen, K. *et al.* Systematic Validation of Protein Force Fields against Experimental Data. **7,** 1–6 (2012).

4. Hess, B., Kutzner, C., van der Spoel, D. & Lindahl, E. GROMACS 4: Algorithms for Highly Efficient, Load-Balanced, and Scalable Molecular Simulation. *J. Chem. Theory Comput.* **4,** 435–447 (2008).

5. Bonomi, M. *et al.* PLUMED: A portable plugin for free-energy calculations with molecular dynamics. *Comput. Phys. Commun.* **180,** 1961–1972 (2009).

6. Bussi, G., Donadio, D. & Parrinello, M. Canonical sampling through velocity rescaling. *J. Chem. Phys.* **126,** 14101 (2007).

7. Bonomi, M. & Parrinello, M. Enhanced Sampling in the Well-Tempered Ensemble. *Phys. Rev. Lett.*

104, 1–4 (2010).

8. Pietrucci, F. & Laio, A. A Collective Variable for the Efficient Exploration of Protein Beta-Sheet Structures: Application to SH3 and GB1. *J. Chem. Theory Comput.* **5,** 2197–2201 (2009).

9. Tiwary, P. & Parrinello, M. A Time-Independent Free Energy Estimator for Metadynamics. *J. Phys. Chem. B* (2014). doi:10.1021/jp504920s
